# Rationale and design of the Children’s Oncology Group (COG) study ALTE1621: a randomized, placebo-controlled trial to determine if low-dose carvedilol can prevent anthracycline-related left ventricular remodeling in childhood cancer survivors at high risk for developing heart failure

**DOI:** 10.1186/s12872-016-0364-6

**Published:** 2016-10-04

**Authors:** Saro H. Armenian, Melissa M. Hudson, Ming Hui Chen, Steven D. Colan, Lanie Lindenfeld, George Mills, Aida Siyahian, Sarah Gelehrter, Ha Dang, Wendy Hein, Daniel M. Green, Leslie L. Robison, F. Lennie Wong, Pamela S. Douglas, Smita Bhatia

**Affiliations:** 1 Department of Population Sciences, City of Hope, 1500, East Duarte Road, Duarte, CA 91010-3000 USA; 2 Department of Epidemiology and Cancer Control, St. Jude Children’s Research Hospital, Memphis, TN USA; 3 Department of Cardiology, Boston Children’s Hospital, Boston, MA USA; 4 Pediatric Cardiology, C.S. Mott Children’s Hospital, Ann Arbor, MI USA; 5 Children’s Oncology Group, Arcadia, CA USA; 6 Survive & Thrive Long-term Follow-up Program, Children’s Mercy Hospital, Kansas City, USA; 7 Division of Cardiology, Department of Medicine, Durham, NC USA; 8 Institute for Cancer Outcomes and Survivorship, University of Alabama at Birmingham, Birmingham, AL USA

**Keywords:** Childhood cancer, Survivors, Cardiomyopathy, Heart failure, Anthracyclines, Risk reduction, Carvedilol

## Abstract

**Background:**

Anthracyclines are widely used in the treatment of childhood cancer. One of the well-recognized side-effects of anthracycline therapy is dose-dependent cardiomyopathy that may progress to heart failure (HF) years after completion of cancer-directed therapy. This study will evaluate the efficacy of low-dose beta-blocker (carvedilol) for HF risk reduction in childhood cancer survivors at highest risk for HF. The proposed intervention has the potential to significantly reduce chronic cardiac injury via interruption of neurohormonal systems responsible for left ventricular (LV) remodeling, resulting in improved cardiac function and decreased risk of HF. The intervention is informed by previous studies demonstrating efficacy in pediatric and adult non-oncology populations, yet remains unstudied in the pediatric oncology population.

**Methods/Design:**

The primary objective of the trial is to determine impact of the intervention on echocardiographic markers of cardiac remodeling and HF risk, including: LV wall thickness/ dimension ratio (LVWT/D; primary endpoint), as well as LV ejection fraction, volume, and blood biomarkers (natriuretic peptides, galectin-3) associated with HF risk. Secondary objectives are to establish safety and tolerability of the 2-year course of carvedilol using: 1) objective measures: hepatic and cardiovascular toxicity, treatment adherence, and 2) subjective measures: participant self-reported outcomes. Two hundred and fifty survivors of childhood cancer (diagnosed <21 years of age), and previously treated with high-dose (≥300 mg/m^2^) anthracyclines will be enrolled in a randomized, double-blind, placebo controlled trial. After baseline assessments, participants will be randomized in a 1:1 ratio to low-dose carvedilol (maximum dose: 12.5 mg/day) or placebo. Carvedilol or placebo is up-titrated (starting dose: 3.125 mg/day) according to tolerability.

**Discussion:**

When completed, this study will provide much-needed information regarding a physiologically plausible pharmacological risk-reduction strategy for childhood cancer survivors at high risk for developing anthracycline-related HF.

**Trial registration:**

ClinicalTrials.gov; NCT02717507

## Background

Anthracyclines (doxorubicin, daunomycin, idarubicin and mitoxantrone) are widely used in the treatment of childhood cancer. The use of these agents has led to significant improvements in the outcome of many childhood cancers, contributing to a growing number of childhood cancer survivors [1]. Current estimates indicate that nearly 60 % of the 450,000 [2] survivors of childhood cancer in the U.S. have been treated with anthracyclines [3, 4]. Clinically, one of the most widely recognized side-effects of anthracycline therapy is dose-dependent cardiotoxicity, manifesting as a continuum from asymptomatic cardiac dysfunction identified by abnormalities of cardiac function/structure on imaging studies, to clinically overt heart failure (HF) and death [1].

The incidence of HF is less than 5 % with cumulative anthracycline exposure of < 300 mg/m^2^; approaches 15 % at doses between 300 and 500 mg/m^2^; and exceeds 30 % for doses > 600 mg/m^2^ [3, 5–8]. A recent report [9] found the adjusted risk of HF after exposure to > 300 mg/m^2^ to be 22-fold (Odds Ratio [OR] = 22.1, *p* < 0.01) higher than those not exposed to anthracyclines, and there are well-established modifiers of this risk such as younger age at exposure and treatment with chest-directed radiation therapy. It is estimated that 1 in 10 children treated with cumulative anthracycline dose exceeding 300 mg/m^2^ will eventually develop anthracycline-related HF [3].

### Rationale for an early intervention

The American College of Cardiology/American Heart Association (ACC/AHA) describe HF as a progressive disorder [10]. Left ventricular (LV) dysfunction begins with injury to, or stress on, the myocardium (Stage A) and is progressive even in the absence of a new identifiable insult to the heart. The subsequent manifestation is asymptomatic change in the LV geometry or structure (Stage B) which precedes overt signs and symptoms of HF (Stages C/D). If left untreated, patients either remain in their current stage or advance to the next stage [10].

In anthracycline-treated childhood cancer survivors, there is a long latency between asymptomatic (Stage A/B) and clinically evident (Stage C/D) disease. Over time, there is a decrease in LV wall thickness (LVWT) and an increase in LV end-diastolic dimension (LVEDD), expressed as decreased LV Wall Thickness/Dimension Ratio (LVWT/D). This decrease in LVWT/D and subsequent increase in LV end-systolic wall stress (ESWS) is a critical component of cardiac remodeling and neurohormonal imbalance that can precede changes in conventional systolic echocardiographic indices such as LV ejection fraction (EF) [6, 11]. Up to 60 % of individuals exposed to high-dose (≥300 mg/m^2^) anthracyclines demonstrate echocardiographic indices of abnormal cardiac remodeling [12] (abnormal LVWT/D). Outcomes are generally poor once an individual develops clinical HF, with 5-year survival rates of less than 50 % [3, 13, 14]. The high prevalence of cardiac dysfunction in patients treated with high-dose anthracyclines, coupled with the poor outcome following onset of HF highlights the importance of early intervention strategies for the prevention of HF in these survivors.

Guidelines recommend angiotensin-converting enzyme (ACE)-inhibitors or β-blockers in individuals with asymptomatic LV systolic impairment (Stage B) [15, 16]. Thus, pharmacologic intervention is standard of care for childhood cancer survivors with Stage B disease [17]. However, initiating treatment after structural changes have been detected (Stage B) may fail to halt progression of cardiac remodeling after anthracycline exposure. Lipshultz et al. [18] reported that while the use of enalapril in Stage B childhood cancer survivors can improve cardiac parameters of remodeling (LV chamber diameter/volume, wall stress), these changes are short-lived, and nearly all patients develop progressive cardiac dysfunction within 6 years of initiation of therapy, supporting the need for intervention at an earlier time point.

Intervention *prior to* LV systolic function impairment has been shown to improve long-term cardiac outcomes in children with Duchene muscular dystrophy (DMD), a population at high risk for dilated cardiomyopathy [19–21]. Much like anthracycline-related cardiomyopathy, cardiac involvement begins as minor echocardiographic abnormalities at a young age, evolves toward LV dilation and subsequent decrease in EF [19]. Treatment of HF with ACE-inhibitors or β-blockers causes transient improvement in cardiac function [8, 20]. However, most develop progressive disease despite intervention (mortality rate: 50 %); due, in part, to the prolonged cardiac remodeling that precedes clinically overt disease, demonstrating the need for an intervention strategy at an earlier time point [19]. The only trial of its kind randomized asymptomatic children with DMD with *preserved* EF (median EF: 65 %) to afterload reduction *v* placebo [20]. Early intervention prevented progression to LV dysfunction, resulting in improved overall 10-year survival in the intervention arm (93 % *v* 65.5 %; *p* < 0.05). This study was one of the first to demonstrate the importance of early intervention in patients with *preserved EF* but at high risk for HF (ACC/AHA Stage A) – a strategy advocated in the current study.

### Rationale for use of low dose carvedilol

There are currently no recommendations for secondary prevention in asymptomatic anthracycline-exposed childhood cancer survivors with preserved EF (Stage A). The only previous clinical trial [22] randomized childhood cancer survivors who were Stage A (using a wide range of anthracycline dose: 75–738 mg/m^2^) to enalapril vs. placebo. The study found that individuals treated with high-dose (300 mg/m^2^) anthracyclines derived the most benefit from the intervention - six out of seven cardiac events occurred in the placebo arm and nearly all were among those treated with high-dose anthracyclines. However, due to the small number of participants in this subgroup (*N* = 37/arm), investigators were unable to make conclusive recommendations regarding prevention of HF with enalapril. Thus, while this study was suggestive of efficacy, there remains a gap in evidence regarding preventive strategies in Stage A childhood cancer survivors at highest risk of HF. Demonstrating treatment efficacy in this setting would result in a paradigm shift in current clinical practice for this growing population of survivors.

With regards to choice of pharmacologic agent (e.g. ACE-inhibitors, β-blockers) to be used in Stage A patients, important lessons can be drawn from prevention studies conducted in high-risk non-oncology populations. In the Carvedilol and ACE-inhibitor Remodeling Mild Heart Failure Evaluation (CARMEN) [23] trial that randomized participants to enalapril or carvedilol, LV remodeling was assessed by serial LV end-systolic volume (LVESV) measurements for 18 months. Carvedilol significantly reduced LVESV (*p* < 0.01) compared to baseline, whereas enalapril only arrested further dilation. Both study arms showed similar safety profiles and withdrawal rates. As a result, it was concluded that while enalapril may have attenuated further myocardial remodeling, carvedilol *reversed* the process, resulting in significant decrease in LVESV and improvement in EF. This improvement in outcome was attributed to the concurrent afterload reduction (α1-blockade) and blockade of adrenergic activation (β1 and β2) uniquely provided by carvedilol. Findings from the CARMEN study highlight the role of third generation beta-blockers such as carvedilol to *reverse* markers of cardiac remodeling [23], making carvedilol a more effective option.

Carvedilol is well-tolerated in children and adolescents with clinical HF [24, 25]. Randomized placebo-controlled clinical trials [24, 25] in children with HF have found comparable rates for adverse events (hypotension, dizziness, fatigue, weakness) and withdrawal between carvedilol and placebo. Importantly, carvedilol has a more favorable safety profile when compared to ACE-inhibitors [26]. Currently, there is a U.S. black-box warning against the use of enalapril (an ACE-inhibitor) in women who are pregnant or planning on becoming pregnant (potential for injury and death to the developing fetus when used after the first trimester) [27]. Carvedilol is a pregnancy category C drug (in the absence of well-controlled studies in humans, potential benefits may warrant its use in pregnant women despite potential risks). Nonetheless, the current trial will not include individuals who are/or planning on becoming pregnant. Overall, the more favorable risk profile of carvedilol provides additional support in favor of carvedilol in the preventive care setting.

The current study proposes using low-dose (12.5 mg) carvedilol to prevent further cardiac remodeling in anthracycline-exposed childhood cancer survivors at high risk for HF; in contrast to the maximum dose used to manage hypertension or HF (~50 mg/day). The selected dose (12.5 mg) reflects an effort to ensure a balance between efficacy and safety /tolerability in a young asymptomatic population with preserved EF. Clinical trials that have provided the rationale for use of a low-dose intervention include: 1) *Prevention trial in children with DMD*: [20, 28] children with preserved EF were randomized to placebo vs. low-dose perindopril (max: 4 mg/day – 50 % lower dose than the maximum [8 mg/day] prescribed for management of hypertension). Low-dose perindopril delayed the progression of LV dysfunction, resulting in improved survival 10 years after the intervention; 2) *Prevention trial in childhood cancer survivors*: [22] Low-dose enalapril (max 10 mg/day) – significantly lower than that used for the treatment of hypertension or LV dysfunction (max 40 mg/day) was efficacious in patients exposed to high-dose anthracyclines; 3) Low-dose carvedilol for management of HF: The Multicenter Oral Carvedilol in Heart Failure Assessment trial [29] evaluated the efficacy of carvedilol *v* placebo for management of patients with mild-moderate HF. Patients randomized to receive 12.5 mg/day had significant improvement in EF from baseline (Δ + 5 % vs. +2 %, *p* < 0.05) and had lower number of mean hospitalizations when compared to placebo (0.14/patient vs. 0.36/patient, *p* < 0.05). A subsequent trial [30] found that doses as low as 5 mg/day can result in improvement in LV EF (Δ +8.7 %) and decrease in cardiovascular disease-related complications when compared to placebo. These studies illustrate that clinically meaningful outcomes can be obtained with low-dose carvedilol such as that proposed in the current trial.

### Rationale for the selected primary and secondary endpoints

LVWT/D, which is analogous to relative wall thickness or mass/volume ratio, was selected as the primary endpoint for the clinical trial because it is a load-independent and objective measure of cardiac remodeling. Change in LVWT/D has been shown to be an independent predictor of sudden cardiac death in children and young adults with cardiomyopathy, making it a clinically important index to monitor for treatment efficacy [31, 32]. Furthermore, abnormally low LVWT/D is the most prevalent cardiac abnormality in survivors of childhood cancer, with a well-established trajectory of change over time (in an un-intervened state) [6, 11], allowing for creating estimates of observable change in the placebo arm over the 2-year study period. Other endpoints such as LVESV and diastolic (LVEDV) volumes as well as EF have been used as intermediate endpoints in clinical trials [33–35] evaluating risk reduction strategies in adults. These indices have well-established prognostic utility in non-oncology populations, and will be included as secondary endpoints for measurement of intervention efficacy.

Natriuretic peptides (NP) such as NT-Pro-BNP and BNP, are established biomarkers for the diagnosis of HF [36], serve as independent risk factors for adverse cardiovascular events, and are being increasingly advocated as objective markers to monitor and adjust anti-congestive treatment [37, 38]. NPs will be included in the longitudinal biomarker assessments for the current study [39]. Galectin-3, a protein produced by activated macrophages, promotes cardiac fibroblast proliferation and collagen synthesis following cardiac injury; [40, 41] Galectin-3 is considered a ‘culprit’ biomarker, analogous to viral load [41]. Serial measurement of galectin-3 may be helpful for monitoring response to pharmacologic intervention. However, whether serial measurements of blood biomarkers could be useful for evaluation of response to pharmacologic intervention remains to be defined. As such, blood biomarkers will serve as secondary endpoints.

Thus, with regards to anthracycline-related HF, the following issues are clear: children exposed to ≥ 300 mg/m^2^ are at high risk of developing cardiomyopathy; if left unattended, asymptomatic cardiomyopathy (Stage B) often progresses to HF; anthracycline-related HF is associated with poor survival; there is a need for a comprehensive intervention approach to halt and reverse the anthracycline-related cardiac damage relatively early in its course in order to prevent the progression to HF. Studies in non-oncology populations have demonstrated safe and effective options for secondary pharmacologic intervention. However, there remains a gap in knowledge regarding the preventive strategies in anthracycline-exposed survivors. The current study will address these gaps in knowledge by evaluating the benefit of pharmacologic intervention targeted to survivors at *highest risk* for developing HF. The proposed intervention with carvedilol may provide a more comprehensive reversal of myocardial remodeling through concurrent sympathetic inactivation (β1,2-blockade) and afterload reduction (α1-blockade), increasing the likelihood of clinically significant risk reduction. This study leverages the established clinical trials network of the Children’s Oncology Group (COG), allowing participation by geographically diverse patient populations. Demonstrating treatment efficacy in this setting has the potential to result in a paradigm shift in current clinical practice for this growing population of childhood cancer survivors.

## Methods/Design

This is a randomized placebo-controlled, double-blind study comparing low-dose carvedilol and placebo for the prevention of LV remodeling in childhood cancer survivors treated with high-dose anthracyclines. Inclusion requirements are as follows: diagnosed with cancer at age ≤21 years, lifetime anthracycline dose: ≥300 mg/m^2^, ≥2 years from completion of therapy, and willingness to provide informed consent. Additionally, male participants must weigh >55 Kg (>10 %-ile for an 18-year old male [CDC]), and females must weigh >50 Kg (>10 %-ile for an 18-year old female [CDC]) at the time of enrollment. Exclusion criteria are as follows: receiving treatment for cardiomyopathy or heart failure; baseline EF <50 % by radionuclide angiogram or echocardiogram or Fraction Shortening <25 % by echocardiogram; uncorrected severe stenotic or regurgitative valvular disease; nondilated (restrictive) or hypertrophic cardiomyopathy or significant systemic ventricular outflow obstruction; sustained or symptomatic ventricular dysrhythmias uncontrolled with drug therapy or implantable device; significant conduction defects (i.e. second or third degree atrio-ventricular block or sick sinus syndrome); bradycardia (heart rate <50 beats per minute); use of an investigational drug or beta adrenergic blockers, including metoprolol, sotalol, within 30 days of enrollment; history of drug sensitivity or allergic reaction to alpha or beta-blockers; history or current clinical evidence of moderate-to-severe obstructive pulmonary disease or reactive airway diseases (i.e. asthma) requiring therapy. Additional exclusion parameters include: abnormal hepatic enzymes (>3 times upper limit of institutional normal); gastrointestinal, or biliary disorders that could impair absorption, metabolism, or excretion of orally administered medications; endocrine disorders (such as primary aldosteronism, pheochromocytoma, hyper- or hypothyroidism) not controlled with medication; insulin dependent diabetes mellitus; anemia (hematocrit <28 %); currently using select CYP2D6 inhibitor or inducer medications; or, inability to swallow pills. Finally, female patients who are pregnant are not eligible; all women of childbearing potential require a negative pregnancy test prior to starting study drug, and be willing to use effective birth control while on study. The current clinical trial has been approved by the Pediatric Central Institutional Review Board (CIRB) and the COG Scientific Council.

## Participants and setting

Eligible patients are identified through retrospective and prospective recruitment at participating COG-member institutions. *Retrospective recruitment strategy*: prior participants in any of the active COG long-term cancer survivorship clinics or associated clinical registries who are alive and eligible based on medical record review are contacted by their respective institutions. Patients who agree to be prescreened are interviewed to confirm eligibility per an established phone scripts. *Prospective recruitment strategy*: the study is introduced to eligible patients at the end of their routine survivorship clinic visit. All participants are enrolled on the study once they have provided written consent, all eligibility requirements for the study have been met, and the patients are registered electronically with the COG Statistics and Data Center.

The current clinical trial has been approved by the Pediatric Central Institutional Review Board (CIRB) and the Children’s Oncology Group (COG) Scientific Council. The CIRB determined that this research satisfies the requirements of 45 CFR 46.405, and 21 CFR 50.52, described as research/clinical investigations involving greater than minimal risk but presenting the prospect of direct benefit to the individual subjects. All adult (≥18 years of age at enrollment) participants will need to provide written informed consent. Children (<18 years of age at enrollment) will need parental signature.

The CIRB has determined that assent of children between the ages of 14–17 is a necessary condition for proceeding with the research (per 45 CFR 46.405, and 21 CFR 50.52). For children younger than 14 years, assent is not a necessary condition for participating in the research (per 45 CFR 46.405, and 21 CFR 50.52), because their capacity is so limited. Principal Investigators should document assent according to local policies and procedures described in their Annual Principal Investigator Worksheet About Local Context.

In all cases, it is expected that investigators will provide children with developmentally appropriate information about the treatment and proposed research participation. In particular, investigators will explain the purpose as well as the design of the clinical trial, risks and benefits of participation, and offer opportunities to ask questions.

## Randomization

Subjects are randomized 1:1 to receive low-dose carvedilol or placebo through a third party drug distributor (Sharp Clinical Services [Phoenixville, PA]). Randomization follows a blocked, stratified randomization design with age at diagnosis (2 strata: <5 years, ≥5 years), time since diagnosis (2 strata: <10 years, ≥10 years), and cardiac irradiation (2 strata: any, none), as stratification factors (8 strata total) and a block size of 4 to balance the number of participants in each arm. Blinded study drug is then assigned to the participant, and the coordinating center is notified that randomization is complete.

## Pharmacotherapy

**Fig. 1:**
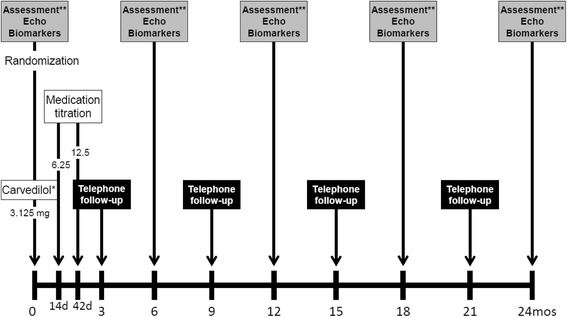
Study procedures

Participants will receive carvedilol or placebo for 2 years. Following a 2-week run-in period with low-dose carvedilol (3.125 mg/day), participants will be up-titrated to a maximum dose of 12.5 mg/day. Those randomized to placebo will receive capsules of identical size, shape, taste and color containing rice flour at the same dosing (up-titration) and schedule. Once an individual achieves his/her maintenance dose (Day 42), they will remain on that dose for a total of 24 months from the date of initial enrollment (Fig. 1).

## Blinding and masking

Participants and the investigative team (e.g. study coordinator, investigators, oncologists, cardiologists) are blinded to group assignment. All echocardiograms will be read independently at the core cardiology lab at the Boston Children’s Hospital, by one reader, blinded to treatment allocation. Clinicians at the participating centers will be provided with global EF measurement in real time, per standard of care.

## Evaluations

After enrollment and randomization, all subjects will be entered into the up-titration period, during which they will receive carvedilol or placebo. The first dose of the double-blind medication is administered at the clinic. The first titration of carvedilol (or matched placebo) occurs 2 weeks following randomization (±2 days), with the subsequent titration occurring 4 weeks later (±7 days). The initial daily dose is 3.125 mg once daily (Dose Level 1), followed by 6.25 mg once daily (Dose Level 2), and 6.25 mg twice daily (Dose Level 3). At each up-titration visit, the following parameters are assessed: interim history and physical examination; sitting pre-dose blood pressure and heart rate; body weight; sitting 1- and 2-hour post-dose blood pressure and heart rate; concomitant medication assessment; and, study medication compliance.

The goal of the up-titration period is to reach the highest dose level that will be continued throughout the maintenance phase. Subjects who are unable to tolerate escalation from dose level 1 to 2 (Day 14) go off protocol therapy. Subjects at dose level 2 can be escalated to dose level 3 or maintained at level 2, depending on tolerability. Dose tolerability will be determined following administration of study drug in clinic. Symptoms are monitored for a minimum of 2 h post medication administration. Individuals who meet the following criteria at the end of the evaluation period will be deemed ineligible for dose escalation: low resting systolic blood pressure (<90 mmHg); bradycardia (heart rate <50 BPM); sustained or symptomatic ventricular dysrhythmias; significant conduction defects (i.e. second or third degree atrio-ventricular block) on electrocardiogram; dizziness: moderate unsteadiness or sensation of movement (Common Terminology Criteria for Adverse Events [CTCAE] v4. Grade 2 toxicity); flushing: moderate symptoms, medical intervention indicated (CTCAE Grade 2).

Participants will enter the maintenance phase receiving the highest dose of study medication that was tolerated in the up-titration phase. This may be dose level 2 or 3. Subsequent return clinic visits will occur on Days 180, 365, 540 and 730 following randomization. The following parameters will be assessed on each visit according to the study observations: interim history and physical examination; echocardiogram; blood draw (cardiac biomarkers, complete blood count, and comprehensive metabolic panel); a symptom log and Health-Related Quality of Life (HRQoL) assessment; and study medication compliance. Interim phone calls with study participants will occur on Days 270, 455, and 630. During the telephone call, there is collection of patient reported symptoms via standardized Symptom Log, medication refill, and assessment of compliance.

After trial completion, study participants will continue clinic follow-up per standard of care, with their first return visit scheduled one year after their Day 730 study visit. All organ function assessments included in the COG survivorship guidelines (www.survivorshipguidelines.org) will be performed, including an echocardiogram. Special attention will be paid to symptoms and signs of cardiac dysfunction on all subsequent annual follow-up survivorship clinic visits. The current COG guidelines recommend annual comprehensive cardiac screening for all survivors who would be eligible for the current study. Information regarding new cardiac events will be reported to the coordinating center as they occur.

## Statistical considerations

We will test our primary hypothesis that carvedilol will be an efficacious option for prevention of cardiac remodeling, as measured by LVWT/D, in childhood cancer survivors treated with high-dose anthracyclines when compared to placebo. Secondary endpoints include LV EF, LV volumes.

LVWT/D z-score, will be determined at three time points (t0, t1 [12 m], t2 [24 m]). Z-scores will be calculated using an established formula [11], and data will be appropriately transformed to normality as necessary. The analysis will be conducted using the Generalized Estimating Equation (GEE) approach for normally distributed data (Proc GENMOD in SAS [Cary, N.C.]). We will use the autoregressive-1 and compound symmetry working covariance matrix structures to account for correlation among repeated measurements within individuals. LVWT/D z-scores will be modeled as a linear function of time. Under the intention-to-treat principle, the efficacy of carvedilol will be examined by comparing the slopes of the longitudinal linear models of LVWT/D z-score between carvedilol and placebo group. The difference in slopes by treatment group will be introduced by including an interaction term of time with group indicator. Treatment effect will be tested by a 1-df test for the interaction. Non-linear effects will be tested by including an interaction of a quadratic term in time with the group indicator. To further examine non-linearity, we will also fit an unstructured mean model, which is a non-parameterized model with four indicator variables of time. Interactions of the time and group indicators will be included to allow for treatment differences at t1, and t2, and their significance tested using a 2-df chi-square test. Because of randomization, we expect no difference in the LVWT/D z-scores at t0 between study arms. However, we will evaluate the success of randomization by examining between-group differences in demographic/clinical characteristics, and mean LVWT/D z-scores at baseline. If imbalance is evident, we will conduct propensity score analysis to adjust for imbalance, to obtain an unbiased estimate of intervention effects [42, 43]. Carvedilol will be considered efficacious if the 1-df test for the time by group interaction in the linear model is significant and the expected LVWT/D z-score is lower for placebo at t1 to t2, indicating the effectiveness of carvedilol in preserving LVWT/D. The analysis will be unadjusted as well as adjusted for covariates (significantly imbalanced covariates and those known to be prognostic for HF: age at cancer diagnosis, chest radiation, and time from diagnosis), will be incorporated in the longitudinal models testing the time by treatment group interaction.

Significance of the carvedilol effects on LVEF and LV volumes will also be evaluated in the manner described above based on testing the significance of the interaction of time by group variables. The distribution of continuous variables will be examined graphically and appropriate transformations made before applying analytical methods based on normal distribution assumption.

Participant drop-out and missing data will be addressed by applying a pattern mixture model [44]. The four possible missing data patterns/strata at t0, t1, t2 (assuming no missing data at t0) will be used as stratification factors in the GEE model to assess the effects of missing data patterns on the estimated treatment effects. Interactions between strata indicators and parameters of treatment effects will be included in the GEE model. A significant interaction indicates that treatment effects vary by missing data patterns and that they should be reported by pattern/strata. An averaged treatment effect (over missing data patterns) will also be calculated [45]. We acknowledge that dropouts may occur differentially between treatment groups for various reasons, possibly resulting in non-ignorable missing data. We intend to collect information on reasons for dropouts, and will apply two analytic approaches to examine the effects of missing data on study results: 1) pattern mixture models and 2) selection models. In addition to intent-to-treat analysis, we will also conduct an as-treated analysis, considering patients’ adherence rate. Instead of the treatment indicator (placebo/carvedilol), we will include adherence rate (0 for placebo; a number between 0 and 1 for carvedilol determined by the adherence rate) in the GEE model.

## Sample size calculations

For sample size calculation, we let Y, the response, be the z-score expected for LVWT/D in both groups. Because of randomization, we expect no difference between the expected values of Y at t0 between treatments. However, if the intervention is efficacious, we expect the linear slopes of the two groups to diverge over time, with no significant change in the slope of LVWT/D for the carvedilol arm. Power/sample size calculation was conducted by assuming a linear change in LVWT/D z-score over time. With this assumption, longitudinal power calculation requires specification of the following: 1) expected values of Y at t0 the carvedilol and control arms, 2) the expected difference in Y at 24 months between the two arms, and 3) the correlations between the repeated measurements over time.

Expected baseline value of LVWT/D was derived from a cross-sectional study evaluating cardiac function in 150 long-term survivors of childhood cancer treated with anthracyclines at City of Hope (median age at evaluation, 24.2 years, time since diagnosis 12.5 years) [46]. Mean LVWT/D z-score for survivors exposed to high-dose anthracycline (≥300 mg/m^2^) was −0.72 (SD 1.0), compared to −0.03 (SD 1.1) in those treated with lower doses (<300 mg/m^2^); *p* < 0.05 [46]. Projected decline in LVWT/D between baseline and 24 months was derived from two clinical trials [11, 47] in children randomized to high-dose anthracycline (300 mg/m^2^) with and without dexrazoxane. These studies were selected because they provided reliable longitudinal data of cardiac function, obtained from multi-institutional clinical trials that used a centralized core echo lab. Patients treated with anthracyclines alone had a decline in LVWT/D Z-score of approximately 0.2/year, providing us with an estimated rate of LVWT/D decline for the placebo arm of the current study. Given these findings, we project that mean LVWT/D z-score will be −0.7 at baseline for both arms of the trial, mean LVWT/D z-score for the placebo arm will decline to −1.1 at the end of the 2-year intervention, and there will be preservation of LVWT/D z-score for the carvedilol arm, resulting in a projected difference at 24 months between the two arms of 0.4.

Since we do not know what the correlation will be between echocardiographic measurements at baseline (t0) and t2, Table 1 depicts minimum detectable differences for correlations ranging from 0.6 to 0.8. In addition, we show detectable differences for important secondary endpoints such as: EF, LVEDV, and LVESV. The longitudinal power calculations were conducted using the program RMASS2 [48]. We assumed a Type I error of 0.05, power of 0.80, 2-sided test, linear trend, and compound symmetry correlation matrix ([ρ] ranging from 0.6 to 0.8). An attrition rate of 15 % per arm/year (voluntary withdrawal, discontinuation due to drug intolerance, unexpected events [pregnancy/relapse]) was assumed. Table 1 shows the minimum effect size at 24 months that can be detected for a given correlation between measures. For example, with 125 individuals in each group, we have 80 % power to detect a difference of at least 0.32 in LVWT/D z-score between the two arms if the correlation between baseline and the 2 year LVWT/D z-score is 0.6. Thus, if the absolute difference between the carvedilol and placebo arms is smaller than 0.4, the current sample size will still be sufficiently powered to detect a lower degree of remodeling prevention. With regards to the secondary endpoints of efficacy, 3–6 mL increases in LVESV, 6–10 mL increases in LVEDV, or 3–5 % drops in EF (from normal resting EF) have been associated with adverse cardiovascular outcomes in clinical trials with patients with preserved or mildly depressed EF [33–35]. Thus, effect sizes presented in Table 1 are plausible and clinically meaningful.

**Table 1:** Detectable differences in study endpoints by echocardiographic correlation estimates between baseline measurement and end of intervention (2 years)

Endpoint	Baseline value	Estimated SD	Correlation between measurements		
			0.6	0.7	0.8
Primary					
LVWT/D (Z-score)	−0.7	1.0	0.35	0.31	0.25
Secondary					
LVEDV (ml)	105.7	26.9	9.4	8.4	6.7
LVESV (ml)	45.0	11.7	4.1	3.6	2.9
LVEF (%)	60.0	5.0	1.8	1.6	1.3

*Abbreviations*: *LVWT/D* left ventricular wall thickness/dimension ratio, *LVEDV* left ventricular end-diastolic volume, *LVESV* left ventricular end-systolic volume, *LVEF* left ventricular ejection fraction, *SD* standard deviation

## Tracking and monitoring of adverse events

The COG Data Safety Monitoring Committee (DSMC) will serve as the DSMC of record. The Protocol Management Team (PMT) consisting of the study Chair, study coordinator, core echo lab Director, collaborating site investigators, site research staff and/or protocol nurse, and the coordinating center statistician will be responsible for monitoring the data and safety of this study, including implementation of any stopping rules for safety and efficacy. Data on adverse events will be reported to the COG DSMC at least annually. However, if, on either arm, two or more cases of the same Grade 3–4 toxicity with probable or definite attributions that do not resolve in 72 h are reported in separate patients, accrual will be held until further review by the COG DSMC to determine whether or not the study should be stopped based on the unblinded data available at that time. Any symptoms that occur while on study will be managed according to good medical practices.

## Discussion

### Methodological considerations

A standardized echocardiographic imaging protocol [49, 50] will be utilized to measure efficacy. This protocol has been successfully implemented in two studies across 22 sites [49, 51], demonstrating low intra-observer %errors for indices selected as endpoints for the current trial: LVWT/D (7.7 %), LVEDV (1.2 %), LVESV (2.3 %), and EF (2.5 %). In these studies, while all core measurements were performed using a single echocardiographic measurement program, the image acquisition at the clinical sites were performed using whatever platform was available at participating centers – a strategy proposed in the proposed clinical trial. For the current study, the Core Lab Director will develop and oversee a program of quality assessment and improvement related to image acquisition (including training of sonographers at each participating center, site certification and adherence to study imaging protocol) and image analysis. The echo machine brand used for the baseline visit will be recorded. In order to control for confounding due to inter-manufacturer variability in measurements, we will request that each subject have all of their echocardiographic studies performed using equipment from the same echo manufacturer. All echocardiograms will be anonymized using established standard operating procedures, and sent to the Core Lab at scheduled time points. Previous studies show that intra-observer variability is half that of inter-observer variability; [49] accordingly, all Core Lab analyses will be performed by a single observer with expertise in pediatric and adult echocardiography.

## Conclusion

There is convincing evidence that childhood cancer survivors treated with high-dose anthracyclines are at risk of developing HF, and that the risk increases with time after treatment. Outcome following onset of clinically overt heart failure is poor, with less than half of all patients surviving an additional 5 years from their HF diagnosis [13]. It is also well-recognized among adult non-oncology populations, that early intervention is critical for reversal of neurohormonal imbalance and markers of cardiac remodeling following ischemic or non-ischemic injury. In addition, among children with genetic predisposition to dilated cardiomyopathy (DMD), pharmacologic intervention has been shown to significantly improve long-term cardiovascular outcomes. There is a paucity of well-conducted randomized interventional trials in childhood cancer survivors at high-risk for HF. Previous studies have shown that while ACE-inhibition may attenuate cardiac remodeling in anthracycline-exposed Stage B childhood cancer survivors, this improvement is short-lived. These studies were limited by their underrepresentation of cancer survivors at highest risk (due to high-dose anthracycline exposure) for HF, small sample size, and use of afterload reduction alone relatively late in the process of cardiac remodeling. In the current study, the combined β1, β2, and α1 blockade provided by carvedilol has the potential to *reverse* markers of early cardiac remodeling in this very high risk population. Given that cardiovascular complications are one of the leading causes of morbidity and mortality in long-term survivors of childhood cancer, we are compelled to evaluate an effective risk-reduction strategy for those at highest risk. Thus, we have developed a pharmacologic intervention for reversal of cardiac remodeling in childhood cancer survivors with a history of prior exposure to high-dose anthracyclines. Findings from this study can potentially be extended to cancer survivors exposed to anthracyclines in adulthood, such as those treated for breast cancer, lymphoma, or sarcoma.

## Abbreviations

ACC/AHA: American College of Cardiology/American Heart Association
ACE: Angiotensin-converting enzyme
CARMEN: the Carvedilol and ACE-inhibitor Remodeling Mild Heart Failure Evaluation
CIRB: Central Institutional Review Board
COG: Children’s Oncology Group
CTCAE: Common Terminology Criteria for Adverse Events
DMD: Duchene muscular dystrophy
DSMC: Data Safety Monitoring Committee
EF: Ejection fraction
ESWS: End-systolic wall stress
GEE: Generalized Estimating Equation
HF: Heart failure
HRQoL: Health-Related Quality of Life
LV: Left ventricular
LVEDD: LV end-diastolic dimension
LVESV: LV end-systolic volume
LVWT: LV wall thickness
LVWT/D: LV wall thickness/ dimension ratio
NP: Natriuretic peptides
OR: Odds ratio
